# Hypoxia-induced TGFBI maintains glioma stem cells by stabilizing EphA2

**DOI:** 10.7150/thno.95141

**Published:** 2024-09-09

**Authors:** Zirong Chen, Junhong Wang, Peng Peng, Guohao Liu, Minhai Dong, Xiaolin Zhang, Yang Zhang, Xue Yang, Lijun Wan, Wang Xiang, Suojun Zhang, Bin Zhang, Qiuxia Wu, Xingjiang Yu, Feng Wan

**Affiliations:** 1 Department of General Intensive Care Unit, Department of Emergency Medicine, The First Affiliated Hospital of Zhengzhou University, Henan Engineering Research Center for Critical Care Medicine, Henan Key Laboratory of Critical Care Medicine, Henan Key Laboratory of Sepsis in Health Commission, Zhengzhou Key Laboratory of Sepsis, Henan Sepsis Diagnosis and Treatment Center, Zhengzhou, China.; 2 Department of Neurosurgery, Tongji Hospital of Tongji Medical College, Huazhong University of Science and Technology, Wuhan, China.; 3 Department of Neurosurgery, Xiangyang Central Hospital, Affiliated Hospital of Hubei University of Arts and Science, Xiangyang, China.; 4 Department of Neurosurgery, Qilu Hospital, Cheeloo College of Medicine and Institute of Brain and Brain-Inspired Science, Shandong University, Jinan, China.; 5 Department of Neurosurgery, The First Affiliated Hospital of Guangxi Medical University, Nanning, China.; 6 Department of Neurosurgery, Guangdong Provincial People's Hospital, Guangdong Academy of Medical Sciences, Guangzhou, China.; 7 Department of Histology and Embryology School of Basic Medicine Tongji Medical College Huazhong University of Science and Technology, Wuhan, China.; 8 Department of Oncology, Tianjin Huanghe Hospital, Tianjin, China.; 9 Department of Physiology, School of Basic Medicine, Tongji Medical College, Huazhong University of Science and Technology, Wuhan, China.; 10 Hubei Key Laboratory of Drug Target Research and Pharmacodynamic Evaluation, School of Basic Medicine, Tongji Medical College, Huazhong University of Science and Technology, Wuhan, China.

**Keywords:** TGFBI, EphA2, GSC, Hypoxia, Microenvironment

## Abstract

**Rationale:** Glioma stem cells (GSCs) have emerged as pivotal drivers of tumor malignancy, sustained by various microenvironmental factors, including immune molecules and hypoxia. In our previous study, we elucidated the significant role of transforming growth factor beta-induced protein (TGFBI), a protein secreted by M2-like tumor-associated macrophages, in promoting the malignant behavior of glioblastoma (GBM) under normoxic conditions. Building upon these findings, the objective of this study was to comprehensively explore the crucial role and underlying mechanisms of autocrine TGFBI in GSCs under hypoxic conditions.

**Methods:** We quantified TGFBI expression in glioma specimens and datasets. *In vitro* and *in vivo* assays were employed to investigate the effects of TGFBI on sustaining self-renewal and tumorigenesis of GSCs under hypoxia. RNA-seq and LC-MS/MS were conducted to explore TGFBI signaling mechanisms.

**Results:** TGFBI is preferentially expressed in GSCs under hypoxic conditions. Targeting TGFBI impair GSCs self-renewal and tumorigenesis. Mechanistically, TGFBI was upregulated by HIF1α in GSCs and predominantly activates the AKT-c-MYC signaling pathway in GSCs by stabilizing the EphA2 protein through preventing its degradation.

**Conclusion:** TGFBI plays a crucial role in maintaining the stem cell properties of GSCs in the hypoxic microenvironment. Targeting the TGFBI/EphA2 axis emerges as a promising and innovative strategy for GBM treatment, with the potential to improve the clinical outcomes of patients.

## Introduction

Glioblastoma (GBM) stands as the most prevalent and aggressive primary CNS tumor in adults, constituting 15% of cases 1. Despite treatment approaches involving extensive surgical resection and adjuvant radio-chemotherapy, patient prognosis remains poor 2. Hypoxia, a hallmark of the microenvironment in various tumors, is strongly associated with a worse prognosis in GBM patients 2-5. Glioma stem cells (GSCs) represent a highly aggressive subset of cancer cells with notable self-renewal and tumorigenic capacities, contributing to disease progression and therapy resistance 2, 6, 7. Nonetheless, the exact molecular mechanisms driving GSCs under hypoxic conditions remain somewhat elusive.

Transforming growth factor beta-induced protein (TGFBI), also known as βig-H3, is an extracellular matrix (ECM) protein that contains four fasciclin-1 domains and a single arginine-glycine-aspartic (RGD) sequence at the C-terminus 8, 9. TGFBI plays a role in a wide array of biological processes, including angiogenesis, migration, invasion, inflammation, adipose metabolism, cell adhesion, and proliferation 8-11. Its upregulation has been observed in various human tumors such as colorectal, breast, prostate, glioma, osteosarcoma, hepatocarcinoma, and ovarian cancer 8, 12, 13. Moreover, increased TGFBI expression in tumors is strongly correlated with poor patient outcomes 8, 12. In our previous research 9, we demonstrated that M2-like tumor-associated macrophages (TAMs) secrete TGFBI, promoting GBM growth through integrin αvβ5-Src-Stat3 signaling, emphasizing the significance of TGFBI in the immune microenvironment of GSCs under normoxic conditions. However, it remains unclear whether and how the autocrine TGFBI under hypoxia impacts on GSCs themselves.

In this study, we investigated the expression and function of TGFBI in GSCs under hypoxic conditions. Our findings revealed an upregulation of TGFBI expression under hypoxia, which significantly decreased upon GSC differentiation. Knockdown of TGFBI resulted in compromised self-renewal and tumorigenic capacities of GSCs within hypoxic microenvironments. TGFBI was identified to interact with EphA2, triggering the activation of downstream signaling pathways in GSCs. These discoveries underscore the critical role of TGFBI in sustaining GSCs under hypoxic microenvironment and propose that targeting the TGFBI/EphA2 axis could offer a promising strategy to address treatment resistance in GBM.

## Methods

### GBM tumor specimens

Human GBM specimens were collected with informed consent from patients between January 2018 to March 2022. A tissue microarray containing 58 representative tissue samples was prepared at Tongji Hospital, Huazhong University of Science and Technology (Table S1). All procedures were conducted in accordance with the principles outlined in the Helsinki Declaration and were approved by the institutional ethics committees (No.TJ-IRB20190315).

### GSC culture and differentiation

The GSCs were cultured in neurobasal-A medium supplemented with B27 (BasalMedia, Cat# S441J7), 10 ng/ml EGF (R&D, Cat#236-EG), 10 ng/ml FGF (R&D, Cat# 4114-TC), penicillin/streptomycin (BasalMedia, Cat# S110JV), 1 mM sodium pyruvate (BasalMedia, Cat# 11360070), and 1 mM L-glutamine (BasalMedia, Cat# 35050061) supplement. For differentiation, GSCs were cultured in serum-containing medium (DMEM medium added with 10% fetal bovine serum, L-Glutamine, and penicillin/streptomycin). The cells were maintained in a humidified incubator at 37 °C with 5% CO2, and for hypoxic conditions, cells were incubated in an atmosphere of 1% O2.

### Orthotopic xenografts

The animal experiments were conducted in accordance with the guidelines and approval of the Institutional Animal Care and Use Committee (IACUC) of Huazhong University of Science and Technology (No. 3984). Four-week-old NU/NU nude mice were obtained from Beijing Vital River Laboratory Animal Technology Co., Ltd. The mice were housed in specific pathogen-free cages with unrestricted access to water and food. For the experiments, 2 × 10^4^ GSCs were injected into the right frontal lobes of anesthetized mice at a depth of 3.5 mm. The manifestation of declining neurologic status, severe weight loss, and physical impairment were considered as clinical endpoints for the animal survival experiments. All surgical procedures were performed under anesthesia via intraperitoneal injection of pentobarbital sodium.

### Immunofluorescence (IF), immunohistochemistry (IHC) and hematoxylin-eosin (HE)

For IF analysis, cells and OCT embedded tumor specimens were fixed in 4% PFA for 30 minutes and permeabilized in PBS containing 0.5% Triton X-100 (Solarbio, Cat#T8200) for 20 minutes. The samples were then blocked with 10% donkey serum at room temperature for 1 hour and incubated with primary antibodies overnight at 4 °C. Subsequently, the samples were incubated with corresponding secondary antibodies for 1 hour at room temperature 14. Nuclear staining was performed using DAPI (Invitrogen). Images were captured using a laser confocal microscope (Olympus, FV1000) and analyzed using Image J software.

For HE and IHC staining, paraffin-embedded GBM tissue or mouse xenograft specimens were deparaffinized and hydrated. Heat-induced antigen retrieval was performed according to the antibody instructions. Incubation with 3% H_2_O_2_ was then carried out. Specimens were subsequently blocked with 5% serum specific to the primary antibody species. They were then incubated with the primary antibody overnight at 4 °C. The following day, sections were incubated with secondary antibodies, followed by DAB detection using horseradish peroxidase (HRP) conjugate and counterstaining of nuclei with hematoxylin. Quantitative analysis was performed by evaluating the Average of density (AOD) using Image J software.

### Immunoblotting (IB)

IB was performed following established protocols. Briefly, cells were lysed using RIPA buffer supplemented with protease and phosphatase inhibitors. Protein samples were separated by SDS-PAGE and transferred onto PVDF membranes. The membranes were then blocked with 5% skim milk. Subsequently, the membranes were incubated with primary antibodies overnight at 4°C, followed by incubation with HRP-conjugated species-specific secondary antibodies. Immunoreactivity was visualized using an ECL kit (Thermo Fisher Scientific, Cat#PI34080), and the analysis was conducted using Image Lab Software.

### Co-Immunoprecipitation (Co-IP)

A total of 3691 GSCs were collected and lysed in Pierce IP Lysis Buffer (Thermo Fisher Scientific, Cat#87788) supplemented with a protease/phosphatase inhibitor cocktail (Thermo Fisher Scientific, Cat#78442,). The lysate was then incubated with the primary antibody or IgG. Protein A/G agarose beads (Santa Cruz, Cat#sc-2003) were added, and then mixture was incubated overnight at 4°C. The precipitated complexs were washed with a wash buffer, boiled with SDS loading buffer, and then subjected to SDS-PAGE and IB. IP was performed at least twice for each cell line.

### Chromatin immunoprecipitation (ChIP) assay

ChIP assays were conducted using the EZ-Magna ChIP A/G Chromatin Immunoprecipitation Kit (Millipore Sigma, Cat#17-10086) following the manufacturer's instructions. Prior to harvesting, GSCs were cultured under hypoxic conditions for 12 hours. For the ChIP reaction, 5 µg of pre-immune rabbit IgG and anti-HIF1α (Novus) antibody were utilized. The relative enrichment of the indicated genes was analyzed by qRT-PCR (Applied Biosystems). The primers utilized for the ChIP-PCR assay at the TGFBI promoter were detailed in Table S2.

### Mass spectrometry assay

A total of 3691 GSCs were collected and lysed in IP lysis buffer. The lysate was incubated with protein A/G agarose and anti-TGFBI antibody (Abclonal, Cat#A11222) or IgG (Abcam, Cat#ab218427) overnight at 4°C. The precipitated complexs were extensively washed with a wash buffer, boiled with SDS loading buffer, and subjected to SDS-PAGE. Gel fragments containing the target peptides were excised for further analysis. The peptides were examined using a Q Exactive HF-X mass spectrometer (Thermo Fisher) and examined using MS/MS (Table S3).

### Cell fraction

The membrane and cytosol fractions were isolated using a specialized kit for protein extraction from membranes and cytosol (Beyotime; P0033). Initially, GSCs were collected and added to an tube containing 1 ml of buffer A. The cells were then lysed until no visible shiny rings around the nuclei were observed. The tube was subsequently centrifuged at 700 g for 10 minutes at 4°C to remove unbroken cells and the nucleus. The resulting supernatant was gently transferred to a new tube and centrifuged at 14,000 g for 10 seconds at 4°C. This supernatant was then transferred to another tube and centrifuged at 14,000g for 10 minutes at 4°C to isolate the cytosol fraction. To obtain the membrane fraction, 200-300 μl of buffer B was added to the precipitate, followed by vigorous vortexing. The tube was then centrifuged at 14,000 g for 5 minutes at 4°C to collect the supernatant containing the membrane fraction.

### Antibodies

Primary antibodies: TGFBI (Abclonal, Cat#A11222, for IB, 1:1000; for IP, 5ug; Proteintech, Cat#10188-AP, for IHC, 1:100; for IF, 1:100), CD133 (Affinity, Cat#BF0403, for IF, 1:100), SOX2 (Proteintech, Cat#66411-1-Ig, for IB, 1:1000; Santa Cruz, Cat#365823, for IF, 1:50; for IHC, 1:50), HIF1α (Proteintech, Cat#20960-1-AP, for IB, 1:1000; for IF, 1:50; for IHC 1:50), carbonic anhydrase 9 (CA9, NOVUS, Cat#NB100-417, for IF, 1:50), OLIG2 (Proteintech, Cat#66513-1-Ig, for IB, 1:1000; for IF, 1:200), Ki67 (Proteintech, Cat#27309-1-AP, for IF, 1:200), EphA2 (Abclonal, Cat#A7183, for IB,1:1000; Santa Cruz, Cat#398832, for IF, 1:50; for IHC, 1:50; for IP, 5ug), Akt (Proteintech, Cat#60203-1-Ig, for IB, 1:5000), phospho-Akt (Ser473) (Proteintech, Cat#66444-1-Ig, for IB, 1:2000; Cell Signaling Technology, Cat#4060, for IF, 1:100; for IHC, 1:50), c-MYC (Proteintech, Cat#67447-1-Ig, for IB, 1:5000; for IF,1:100; for IHC, 1:100), phospho-EphA2 (Y588) (Proteintech, Cat#30263-1-AP, for IB, 1:1000), Integrin αV (Proteintech, Cat#27096-1-AP, for IB, 1:1000), Integrin β5 (CST, Cat#3629, for IB, 1:1000), Notch1 (Cell Signaling Technology, Cat#3608, for IB, 1:1000). Species-specific antibodies for IB: Anti-rabbit IgG (Biosharp, Cat#1:5000), anti-mouse IgG (Biosharp, Cat#1:5000). Species-specific antibodies for IF: Anti-Rabbit IgG (Invitrogen, Cat#7074P2, 1:200), anti-mouse IgG (Invitrogen, Cat#2266877, 1:100).

### Plasmid generation and lentiviral vector construction

For shRNA plasmid construction, the human TGFBI and HIF1α-specific coding sequences were cloned into the pLKO.1-puro vectors (Sigma-Aldrich, Cat#SHC002). The overexpression plasmid for human TGFBI and EphA2 were created by cloning their coding sequences into the PLVX-puro-3 vectors. To produce lentiviruses, packaging vectors psPAX2 (Addgene), and p-CMV-VSV-G (Addgene) were co-transfected with lentiviral vectors carrying control shRNA, specific shRNA, or TGFBI and EphA2 into 293T cells. Lentivirus production and transduction were performed as described previously 5, 14.

### Cell viability assays

Cell viability assays were performed following established protocols 14. In brief, 1×10^3^ cells were seeded into individual well of 96-well plates with 6 repetitions and cultured under hypoxic conditions. After the indicated days, cell viability was assessed using the CellTiter-Lumi™ Plus Assay reagent (Beyotime, Cat# C0068). The results were normalized to day 0, presented as mean ± standard deviation.

### Tumor sphere formation assays and *in vitro* limiting dilution assay

For tumor sphere formation, GSCs were seeded into 24-well plates at a density of 1,000 cells per well and cultured under hypoxic conditions for 4 days. The quantification of tumorspheres formed was assessed subsequently. In the *in vitro* limiting dilution assay, GSCs were seeded at varying densities (1, 5, 10, 20, 40 cells per well) into a 96-well plate with six replicates for each density. The efficiency of sphere formation was calculated for each group 9.

### 5-Ethynyl-2′-deoxyuridine (EdU) incorporation assay

EdU assays were conducted utilizing the Cell-Light EdU Apollo567 *in vitro* Kit (RiboBio, Cat# C103101) following the manufacturer's instructions. Cells were seeded into a 24-well plate with three replicates per group. Following a two-hour incubation with 50 μM EdU, cells were fixed in 4% paraformaldehyde and stained with Apollo Dye Solution. Nucleus were stained with DAPI. Images were acquired using a laser confocal microscope (Olympus, FV1000), and the count of EdU-positive cells was quantified.

### *In vitro* ubiquitylation assay

GSCs were exposed to 5µM MG132 (MCE, Cat#133407-82-6) or a vehicle control for 6 hours prior to collection. The cells were then washed with cold PBS and lysed using RIPA buffer through sonication. The lysates were then subjected to IP using 5µg of anti-EphA2 antibody (Santa Cruz, Cat#398832) and further analyzed via western blotting. Anti-Ubiquitin antibody (Cell Signaling Technology, Cat#43124, 1:1000) or anti-EphA2 antibody (Abclonal, Cat#A7183, 1:1000) were untilized to detect the ubiquitylation of EphA2. The ubiquitylation assay was performed three times for each cell line.

### RNA isolation and quantitative real-time PCR (qRT-PCR)

The total RNA was isolated using Trizol (Invitrogen) and then reverse transcribed to cDNA with the HiScript II Q RT SuperMix (Vazyme, R223-01) following the manufacturer's instructions. PCR amplifications were performed using ChamQ SYBR Master Mix (Vazyme, Q311-02/03). The primer pairs for qRT-PCR were detailed in Table S2.

### Bioinformatics analysis

The glioma datasets, including RNA-seq and clinical information, sourced from The Cancer Genome Atlas (TCGA) and Chinese Glioma Genome Atlas (CGGA), were obtained from GlioVis (http://gliovis.bioinfo.cnio.es/). The GSE86237 dataset was downloaded from Gene Expression Omnibus (GEO) (https://www.ncbi.nlm.nih.gov/geo/). For the analysis of potential HIF1A binding sites within the TGFBI promoter, the JASPER website (http://jaspar.genereg.net/) was consulted for the binding motif 15.

### Statistical analysis

All statistical analyses were conducted using the R software package (version 4.0.2) or GraphPad Prism (version 8.0.2). The summary data were presented as mean ± SD. The unpaired Student's t-test was used for comparing two groups, while one-way ANOVA was employed for comparisons involving multiple groups. Additionally, Kaplan-Meier curves were generated and compared using the Log-rank test. *P* < 0.05 was considered statistically significant.

## Results

### TGFBI correlates with glioma malignancy and hypoxic microenvironment

TGFBI exhibits commonly upregulated in human GBM. To explore the role of TGFBI in gliomas, we conducted an analysis using the CCGA and TCGA databases. We found that TGFBI expression is higher in IDH wild-type (IDH wt) gliomas in comparison to IDH mutant gliomas (Figure S1A). Moreover, within the subset of IDH wt gliomas, higher TGFBI expression levels were correlated with worse patient survival (Figure S1B). To delve deeper, we assessed TGFBI expression across various glioma grades. Our examination of 58 human samples, encompassing normal (n = 11), grade II glioma (n = 11), grade III glioma (n = 7), and GBM (n = 29), highlighted a notable increase in TGFBI expression in high-grade tumors (Figure S1C), suggesting a potential role for TGFBI in driving glioma malignancy.

**Figure 1:**
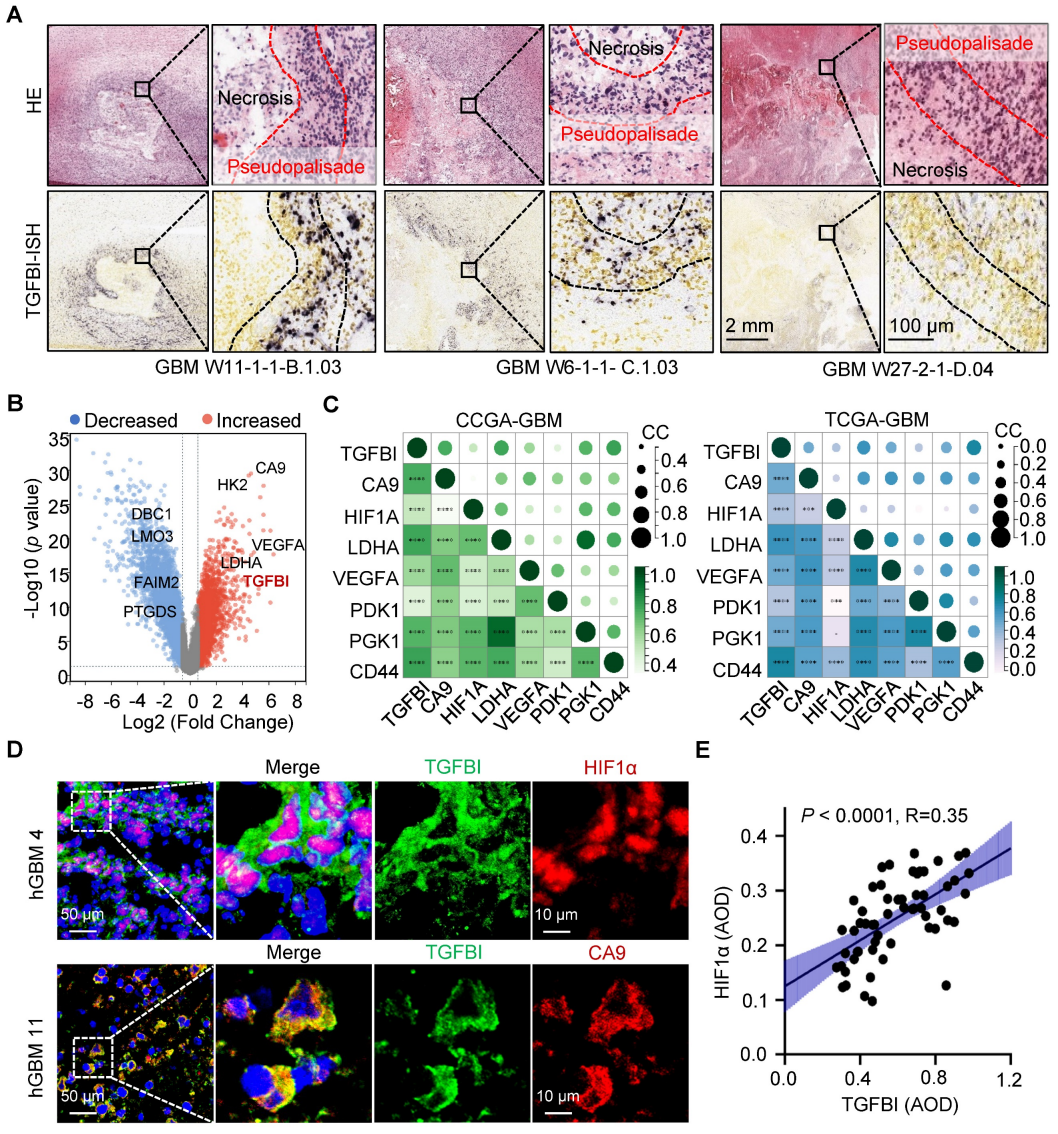
TGFBI is associated with the hypoxic microenvironment in human gliomas. (A) FISH staining of TGFBI in human GBM specimens (Ivy gap datasets). Scale bars: 2 mm; enlarged image: 100 μm. (B) Volcano map showing genes highly expressed in hypoxia region (pseudopalisade and microvascular proliferation regions). Each dot represents a gene. (C) Correlation between TGFBI and mRNA expression of hypoxia-related gene in the CCGA-GBM and TGGA-GBM datasets. CC, correlation coefficient; Dot size and color represent the correlation coefficient. (D) IF staining of TGFBI (green) and two hypoxia-associated markers, HIF1α (above, red) and CA9 (bottom, red), in human GBM specimens. Scale bars: 50 μm; enlarged image: 10 μm. (E) IHC staining demonstrating the association between TGFBI and HIF1α proteins in human gliomas. AOD, Average of density; n = 58.

Additionally, analysis of the Ivy Glioblastoma Atlas Project datasets indicated a preferential expression of TGFBI in cells localized within the pseudopalisading region, known for its hypoxic characteristics (Figure 1A) 16. Further investigation of TGFBI expression in normoxic and hypoxic regions, specifically the pseudopalisading and microvascular proliferation regions, demonstrated upregulation of TGFBI under hypoxic conditions (Figure 1B). Moreover, our analysis of the CGGA and TCGA databases revealed a positive correlation between TGFBI expression and several hypoxia markers, including CA9, HIF1A, LDHA, VEGFA, PDK1, PGK1, and CD44 (Figure 1C). To explore the correlation between TGFBI expression and the hypoxic microenvironment in human GBM specimens, we assessed the protein expression of TGFBI, HIF-1α, and CA9. Our results indicated predominant TGFBI expression in tumor cells positive for HIF1α and CA9 (Figure 1D). Additionally, IHC staining of TGFBI expression in 58 glioma specimens revealed a positive correlation between TGFBI and HIF1α (Figure 1E). Notably, within the subset of IDH wt gliomas in the CGGA and TCGA databases, higher levels of HIF1α and CA9 expression were linked to worse patient survival (Figure S1D-E). These findings suggest that TGFBI expression is associated with hypoxia in GBM and could play a role in the progression of GBM malignancy under hypoxic conditions.

### HIF1α induces the expression of TGFBI in GSCs under hypoxia

**Figure 2:**
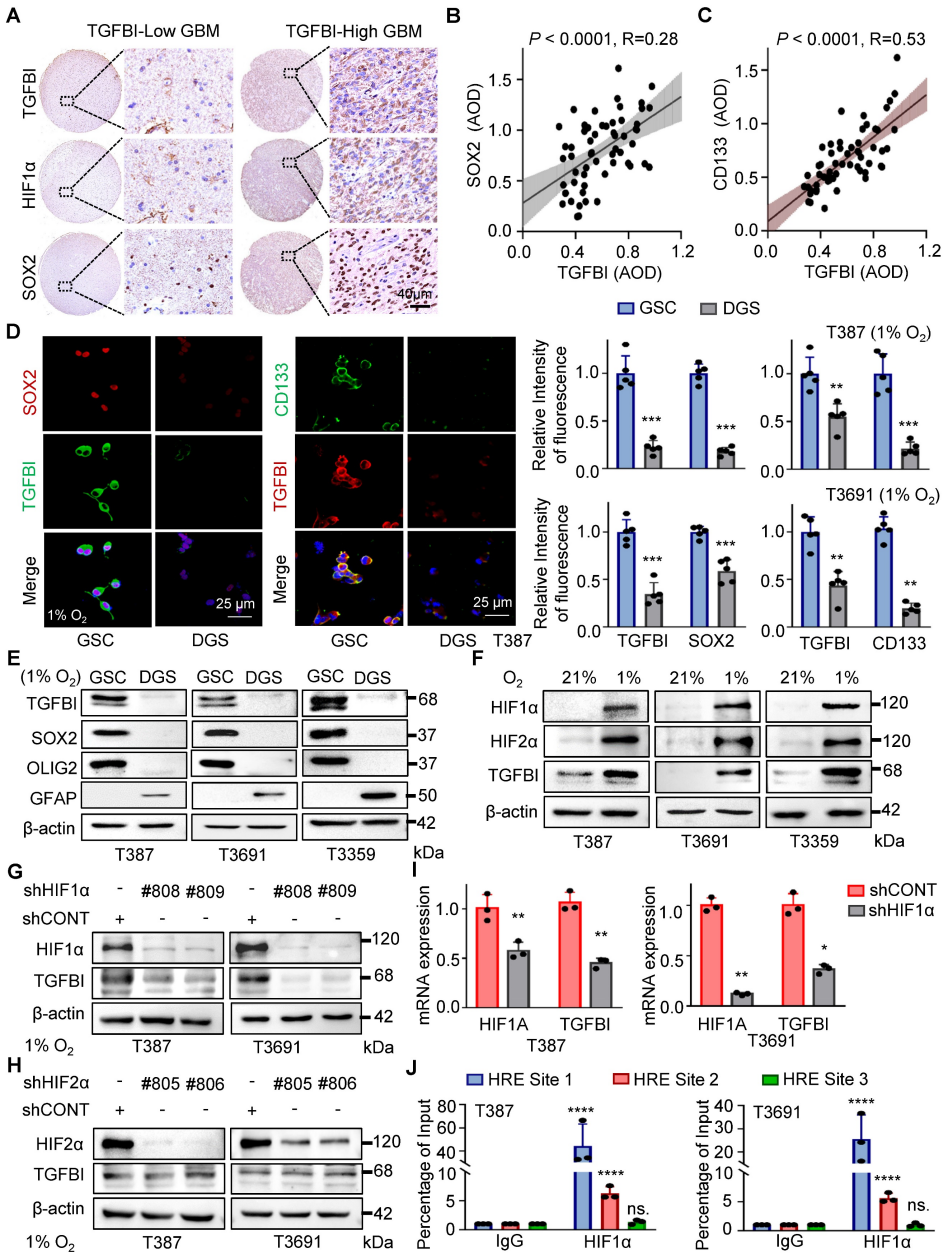
TGFBI induction by HIF1α in GSCs under hypoxia. (A) IHC staining of TGFBI, HIF1α, and SOX2 in the same human high- and low-expression GBM tissues. Scale bars: 40 μm (B) IHC staining demonstrating the association between TGFBI and SOX2 proteins in human gliomas. n = 58 (C) IHC staining demonstrating the association between TGFBI and CD133 proteins in human gliomas. n = 58 (D) IF staining of TGFBI and two stem cell-associated markers (SOX2, CD133) in T387 GSCs. Also shown is the quantification of the relative intensity of fluorescence of T387 and T3691 GSCs (right, n = 5). (E) IB of TGFBI, SOX2, OLIG2, and GFAP proteins in the indicated GSCs and differentiated GSCs (DGS). (F) IB of TGFBI, HIF1α, and HIF2α in matched GSCs cultured under standard (21% O_2_) or hypoxic (1% O_2_) conditions for 24 hours. (G) IB of TGFBI protein expression in the GSCs transduced with shCONT or shHIF1α and (H) shHIF2α under hypoxia. (I) qRT-PCR analysis showing mRNA expression of TGFBI and HIF1α in GSCs transduced with shCONT or shHIF1α. (J) ChIP analyses showing HIF1α binding to the TGFBI promoter in GSCs under hypoxia. Data are presented as the mean ± SD. **P <0.01; ***P < 0.001; ****P < 0.0001.

In addition to HIF1α, we observed a correlation between TGFBI expression and SOX2, a key transcription factor in GSC self-renewal (Figure 2A). Analysis of an independent transcriptome database (GSE86237) also indicated elevated TGFBI expression in CD133^+^ tumor cells compared to the tumor bulk (Figure S2A). Further IHC analysis of human glioma specimens indicated a positive correlation between TGFBI and SOX2 (Figure 2B) expression level, as well as CD133 (Figure 2C).

It's well-documented that stem cells are enriched in hypoxic niches, and tumor hypoxia sustains the stem cell phenotype 3, 14. To investigate whether TGFBI is induced in GSCs under hypoxia, we differentiated GSCs and found that TGFBI was notably expressed in GSCs but not in matched differentiated cells (Figure 2D-E and Figure S2B). Furthermore, we confirmed that GSCs can upregulate TGFBI under hypoxic conditions (Figure 2D-I).

Given the involvement of the transcription factor HIF in regulating most hypoxia-responsive genes, we predicted three potential HIF1α binding sites according to the JASPAR website (Figure S2C). To validate the role of HIF in TGFBI regulation, utilized shRNA to knock down HIF1α and HIF2α. Under hypoxic conditions, the downregulation of HIF1α, but not HIF2α, resulted in decreased TGFBI expression in GSCs (Figure 2G-H). Additionally, the reduction of HIF1α led to a decrease in TGFBI mRNA expression (Figure 2I). ChIP analysis further confirmed the binding of HIF1α to the TGFBI promoter (Figure 2J). Overall, these findings suggest that HIF1α plays a crucial role in the predominant expression of TGFBI in GSCs under hypoxic conditions.

### TGFBI maintains the self-renewal and tumorigenic potential of GSCs

**Figure 3:**
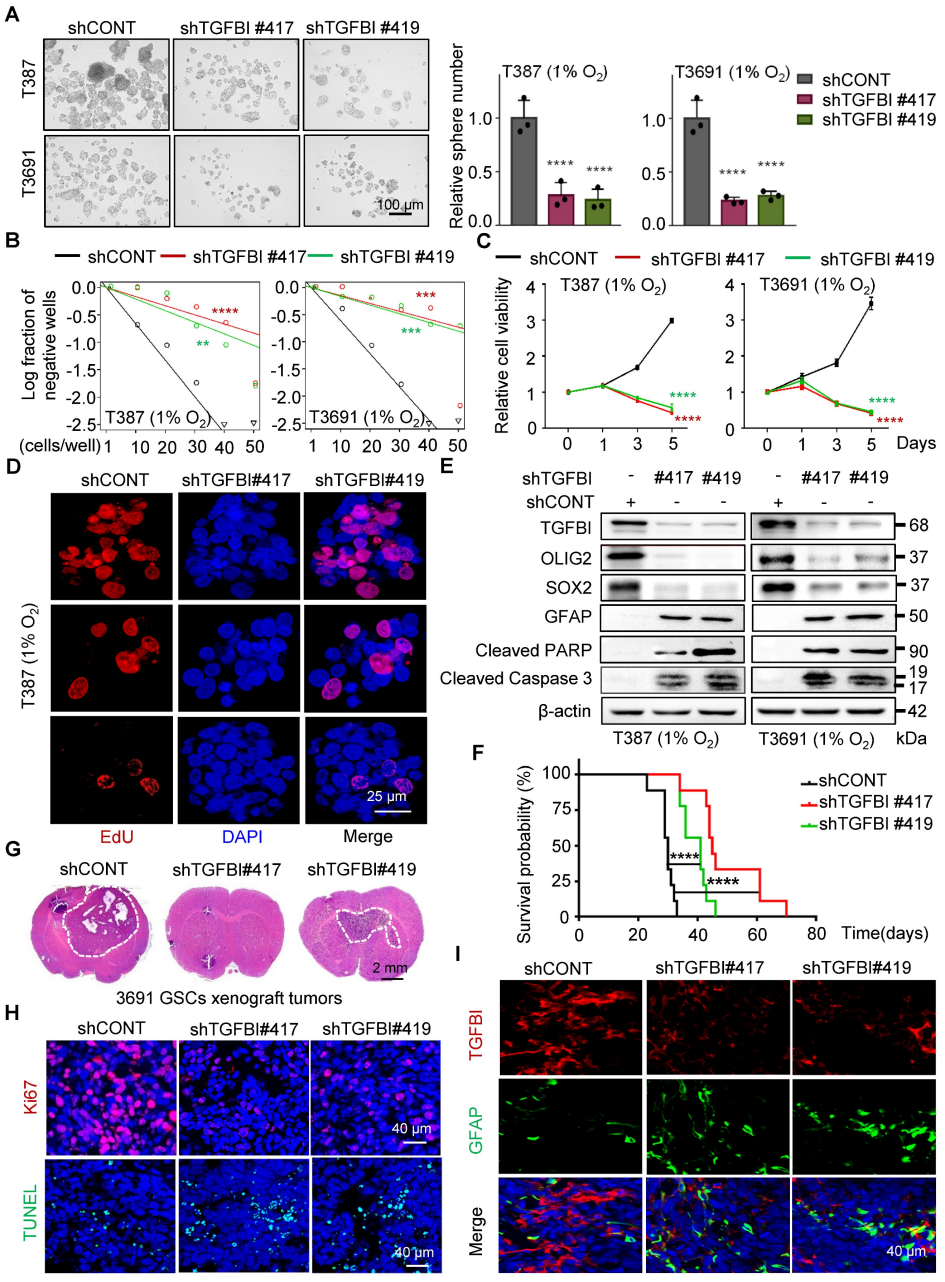
TGFBI plays essential role in maintaining self-renewal and tumorigenesis of GSCs. (A) Bright-field microscopy (left) showing the tumorsphere formation of GSCs transduced with shCONT or shTGFBI. Quantification of relative tumorsphere numbers is depicted (right, n = 3). Scale bars: 100 μm. (B) Limiting dilution assays of indicated GSCs. (C) Cell titer assay of indiated GSCs. n = 6 (D) Confocal image of EdU incorporation in T387 GSC tumorspheres. n = 4; EdU is represented in red. The image of T3691 and quantification of the fraction of EdU^+^ cells is avaliable in Figure S3A. Scale bars: 25 μm (E) IB of stem-related (SOX2, OLIG2, and GFAP) and apoptosis-related (cleaved PARP and cleaved caspase3) proteins in the GSCs transduced with shCONT or shTGFBI. (F) Kaplan-Meier survival curve of mice bearing T3691 GSCs expressing shCONT or shTGFBI. n = 10 (G) H&E staining of brain sections from xenograft mice. Scale bars: 2 mm. (H) IF image in mouse xenografts injected with shCONT and shTGFBI GSCs. Ki67 is shown in red, TUNEL in green. Scale bars: 40 μm. Quantification of the fraction of Ki67^+^ and TUNEL^+^ cells in mouse xenografts are shown in Figure S3B. n = 5 (I) IF image of TGFBI and GFAP in mouse xenografts. Scale bars: 40 μm. Quantification of the relative intensity of fluorescence is shown in Figure S3C. n = 4; Data are presented as the mean ± SD. **P < 0.01; ***P <0.001; ****P < 0.0001.

To investigate the functional role of TGFBI in GSCs, we suppressed TGFBI expression in GSCs under hypoxic conditions. Through a series of *in vitro* assays assessing self-renewal capacity, we observed a significant impairment in sphere formation, cell viability, and proliferation capacity of GSCs upon TGFBI inhibition (Figure 3A-D and Figure S3A). Moreover, we noted a significant decrease in the expression of self-renewal markers (SOX2 and OLIG2) following TGFBI inhibition, accompanied by an increase in the differentiation-associated marker GFAP. Additionally, inhibiting TGFBI in GSCs let to an upregulation of apoptosis-related proteins (cleaved PARP and cleaved caspase 3) (Figure 3E).

*In vivo*, examination of orthotopic xenografts of GSCs revealed that mice injected with shTGFBI GSCs experienced significantly prolonged survival compared to those injected with shCONT GSCs (Figure 3F). Furthermore, while mice injected with shCONT displayed neurological signs or clinical deterioration, mice injected with shTGFBI remained asymptomatic with smaller or absent tumors (Figure 3G). The tumors originating from shTGFBI mice exhibited decreased proliferation (Ki67) and increased levels of apoptosis (Figure 3H and Figure S3B), as well as heightened differentiation levels (Figure 3I and Figure S3C), aligning with the *in vitro* findings. Altogether, these results demonstrate the crucial role of TGFBI in maintaining the self-renewal and tumorigenic potential of GSCs under hypoxic conditions.

### TGFBI facilitates the AKT-c-MYC pathway to play a role in GSC maintenance

**Figure 4:**
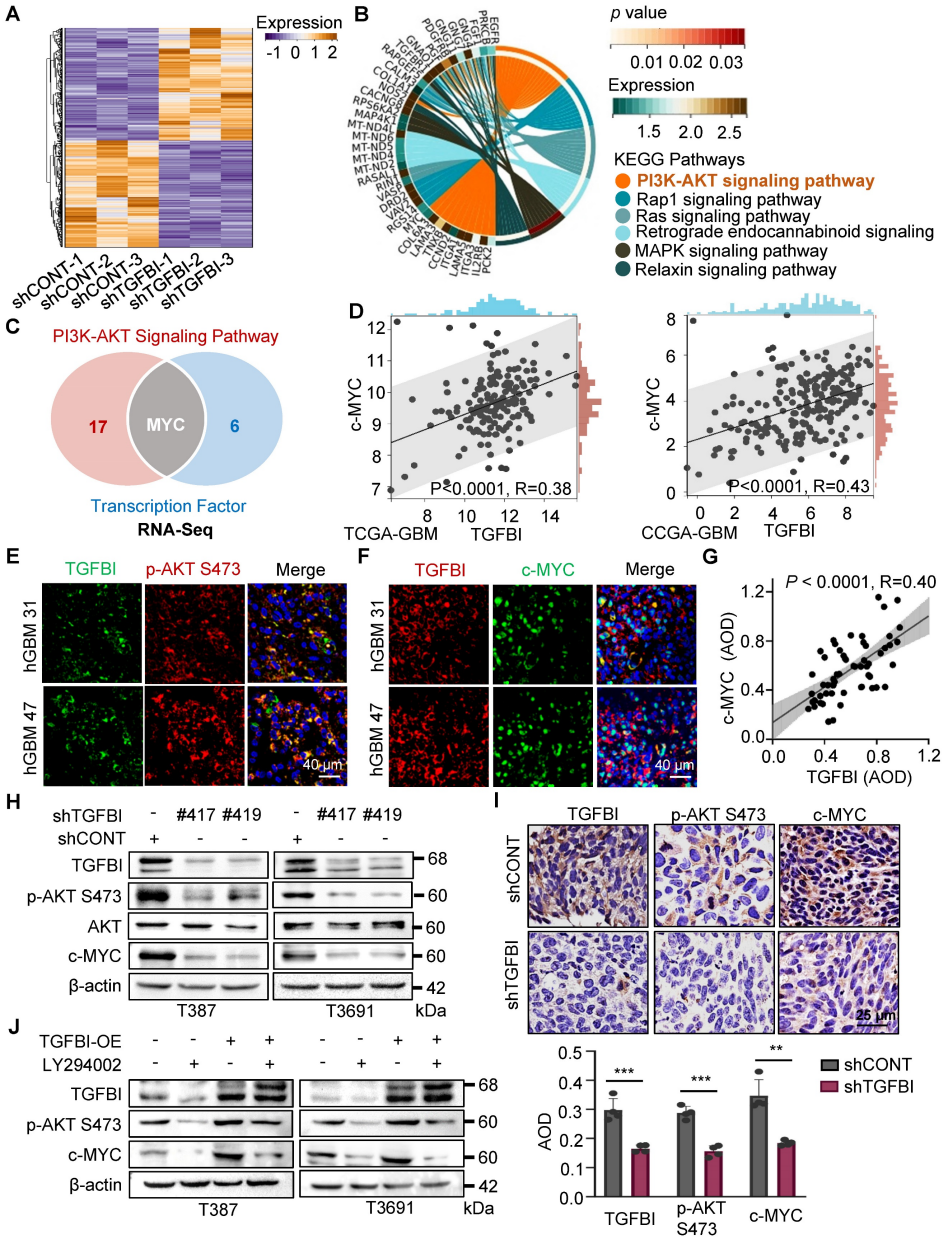
TGFBI sustains GSCs through AKT-c-MYC pathway. (A) Heatmap illustrating the transcriptional profile of T3691 GSCs transduced with shCONT or shTGFBI. (B) KEGG pathway analysis of downregulated differentially expressed genes in shTGFBI T3691 GSCs (compared to the shCONT group). (C) Venn diagram depicting the differentially expressed genes highly active in the PI3K-AKT signaling pathway and serving as transcription factors. (D) Scatter plot displaying the correlation between TGFBI and c-MYC mRNA expression in the TCGA-GBM and CGGA-GBM datasets. (E) IF image of TGFBI and p-AKT S473 and (F) c-MYC in human GBM specimens. Scale bars: 40 μm. (G) IHC stain demonstrating the association between TGFBI and c-MYC proteins in human gliomas. n = 58 (H) IB of p-AKT S473 and c-MYC proteins in the GSCs transduced with shCONT or shTGFBI under hypoxia. (I) IHC stain of TGFBI, p-AKT S473, and c-MYC proteins in the mouse xenografts. Also shown is the quantification of the average density of mouse xenografts (bottom, n = 4). (J) IB of p-AKT S473 and c-MYC proteins in the indicated cells. LY294002, an AKT pathway inhibitor.

To elucidate the mechanisms through which TGFBI regulates the self-renewal and tumorigenesis of GSCs, we performed next-generation transcriptional sequencing on T3691 GSCs treated with either shCONT or shTGFBI under hypoxia (Figure 4A). Subsequently, we conducted a Kyoto Encyclopedia of Genes and Genomes (KEGG) pathway analysis on the downregulated differentially expressed genes (DEGs), revealing that TGFBI deficiency impaired the AKT signaling pathway in T3691 GSCs (Figure 4B). Among these downregulated DEGs, we identified 7 transcription factors (c-MYC, OLIG2, BATF3, ELK3, TEAD1, AKNA, and HLTF) that could potentially be influenced by TGFBI and play a role in regulating the self-renewal and tumorigenesis of GSCs. Further investigation indicated that c-MYC might be a potential transcription factor regulated by TGFBI through the AKT signaling pathway (Figure 4C). Notably, c-MYC has been reported to promote self-renewal and tumorigenesis of stem cells while maintain their undifferentiated state 17, 18.

To assess whether TGFBI impacts GSCs through the AKT-c-MYC signaling axis, we analyzed the TCGA and CGGA GBM databases. Correlation analysis revealed a positive relationship between TGFBI and c-MYC expression level (Figure 4D). Subsequently, IF staining on human GBM samples demonstrated co-expression of TGFBI with phosphorylated AKT at Ser473 (p-AKT S473) and its downstream target, c-MYC (Figure 4E-F). Furthermore, IHC staining on human glioma specimens confirmed a positive correlation between TGFBI and c-MYC level (Figure 4G).

*In vitro* assays also revealed that shTGFBI GSCs exhibited decreased expression of p-AKT S473 and c-MYC compared to their shCONT counterparts (Figure 4H). Consistent with these *in vitro* results, inhibition of TGFBI expression led to decreased expression of p-AKT S473 and c-MYC in orthotopic GBM xenografts compared to the shCONT group (Figure 4I). Treatment with LY294002, an AKT pathway inhibitor, reduced the protein expression of p-AKT S473 and c-MYC, and this effect could be rescued by TGFBI overexpression (Figure 4J). Collectively, these findings demonstrate that TGFBI maintains GSCs through the AKT-c-MYC signaling pathway under hypoxic conditions.

### The binding of TGFBI to EphA2 enhances EphA2 stability by suppressing the ubiquitin-proteasome pathway

**Figure 5:**
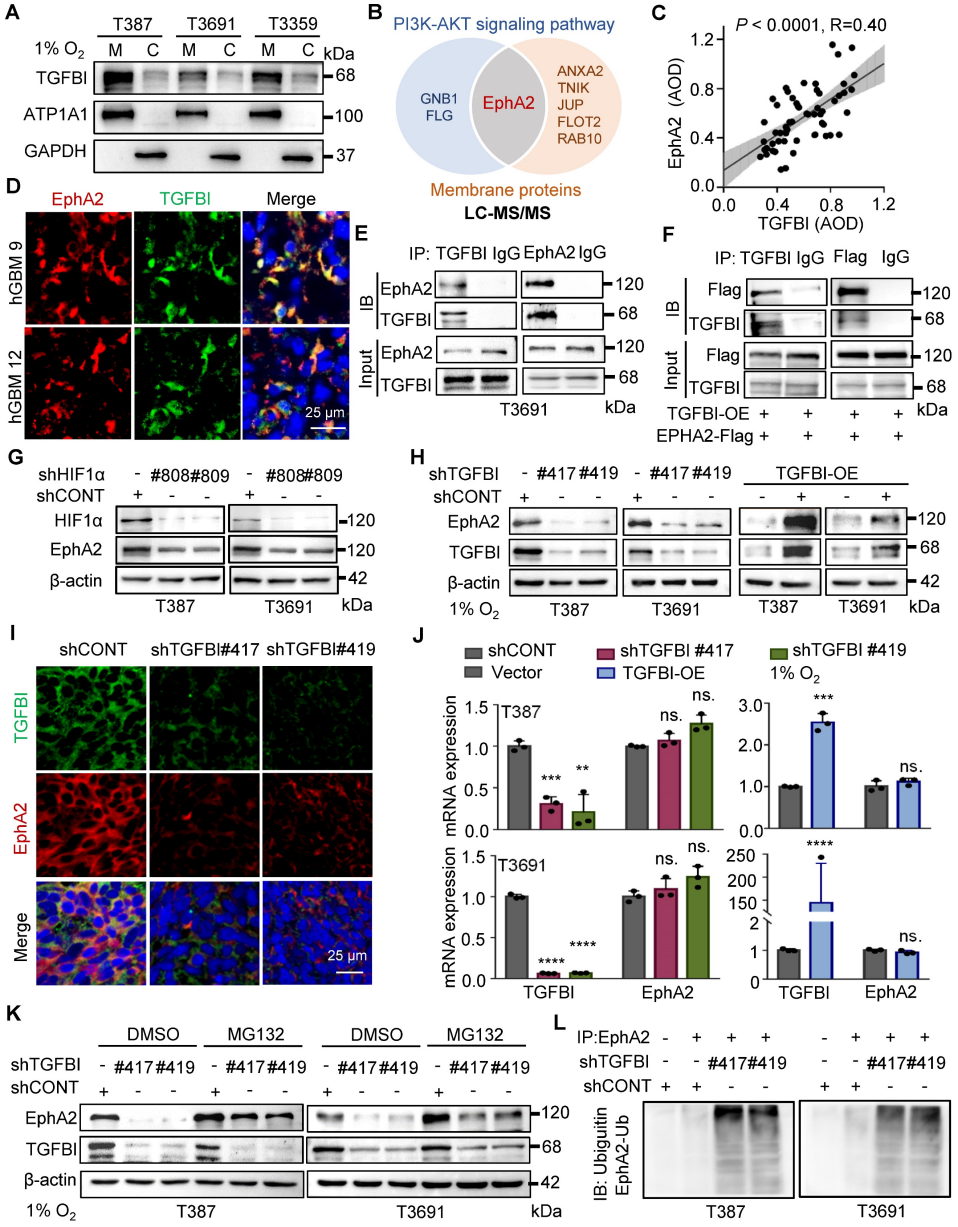
TGFBI binds to EphA2 and stabilizes it by inhibiting its proteasomal degradation. (A) Analysis of membrane and cytosol protein extraction in GSCs under hypoxia. M, membrane; C, cytosol (B) Venn diagram displaying TGFBI-binding proteins of T3691GSCs under hypoxia highly expressed in the PI3K-AKT signaling pathway and located in membrane according to LC-MS/MS. (C) IHC staining demonstrating the correlation between TGFBI and EphA2 proteins in human gliomas. n = 58 (D) IF image of TGFBI and EphA2 in human GBM specimens. Scale bars: 25 μm. (E) Co-IP of endogenous TGFBI and EphA2 in T3691 GSCs under hypoxia. IgG served as a control. (F) Co-IP of exogenous TGFBI and EphA2 in 293T. (G) IB of EphA2 protein in the GSCs transduced with shCONT or shHIF1α under hypoxia. (H) IB of EphA2 protein in the GSCs with TGFBI knockdown or overexpression. (I) IF image of TGFBI and EphA2 in the indicated mouse xenografts. The quantification of the relative intensity of fluorescence is shown in Figure S4F (n = 8). Scale bars: 25 μm. (J) qRT-PCR analysis of TGFBI and EphA2 mRNA expression in the indicated GSCs. (K) IB of EphA2 proteins in the indicated cells. MG132, a proteasome inhibitor. (L) Co-IP of ubiquitin-EphA2 in the indicated GSCs. The input is shown in Figure S4H. Data are presented as the mean ± SD. ns., no significance; **P < 0.01; ***P < 0.001; ****P < 0.0001.

To investigate the regulatory role of TGFBI in the intracellular AKT-c-MYC signaling pathway of GSCs, we initially conducted membrane and cytosol protein extractions from GSCs under hypoxia (Figure 5A). We also conducted co-staining of TGFBI with membrane-localized proteins (human HLA-A and CD133, Figure S4A-B) in mouse xenografts to elucidate the subcellular localization of TGFBI. Our findings demonstrated that TGFBI predominantly localizes at the cell membrane. Subsequently, we employed IP and mass spectrometry to identify potential binding partners of TGFBI derived from GSCs under hypoxia, leading to the identification of EphA2 as a candidate binding partner involved in the AKT signaling pathway and membrane localization (Figure 5B). The expression of EphA2 in GSC spheres was confirmed through IF (Figure S4C), and a correlation between TGFBI and EphA2 in human glioma specimens was observed via IHC staining (Figure 5C). Notably, obvious co-localization of TGFBI and EphA2 was evident in a human GBM sample through IF (Figure 5D). The interaction between TGFBI and EphA2 was further authenticated through co-IP in GSC lines (Figure 5E and Figure S4D). *In vitro* IP conducted in the 293T cell line reaffirmed the binding between TGFBI and EphA2 (Figure 5F). Additionally, our research suggested that EphA2 exhibit a significantly upregulation under hypoxia compared to integrins, which were identified as the primary binding partner of TGFBI under normalxia conditions (Figure 5G, Figure S4E) 9. Consistently, knockdown and overexpression of TGFBI led to alterations in the expression of EphA2 protein (Figure 5H). IF analysis of mouse xenografts revealed that TGFBI inhibition decreased EphA2 levels in the tumor (Figure 5I and Figure S4F). Consequently, we proceeded to investigate the potential regulatory mechanism of EphA2 by TGFBI.

Initially, we examined the mRNA expression level of EphA2 following regulating TGFBI. The qRT-PCR results showed that mRNA expression of EphA2 remained stable when the expression of TGFBI was altered (Figure 5J). Given the significant impact on EphA2 protein levels, we hypothesized that TGFBI binds to EphA2 to hinder its protein degradation. To test this hypothesis, we treated GSCs with inhibitors targeting the lysosome and proteasome protein degradation pathways, chloroquine and MG132, respectively. Treatment with MG132 resulted in an increase in EphA2 levels, while treatment with chloroquine did not induce noticeable changes. Additionally, treatment with MG132 partially rescued the EphA2 levels affected by TGFBI inhibition. However, treatment with the lysosome inhibitor chloroquine failed to restore EphA2 levels upon TGFBI knockdown but obviously increased Notch1, serving as a positive control of chloroquine (Figure 5K and Figure S4G) 14, 19. Furthermore, IP analysis indicated significant increase of ubiquitinated EphA2 in GSCs following TGFBI knockdown under hypoxia (Figure 5L and Figure S4H). Overall, these findings suggest that TGFBI primarily binds to EphA2 and regulates its degradation in GSCs under hypoxic conditions.

### The binding of TGFBI to EphA2 promotes the self-renewal of GSCs and tumorigenesis *in vitro* and *in vivo*

**Figure 6:**
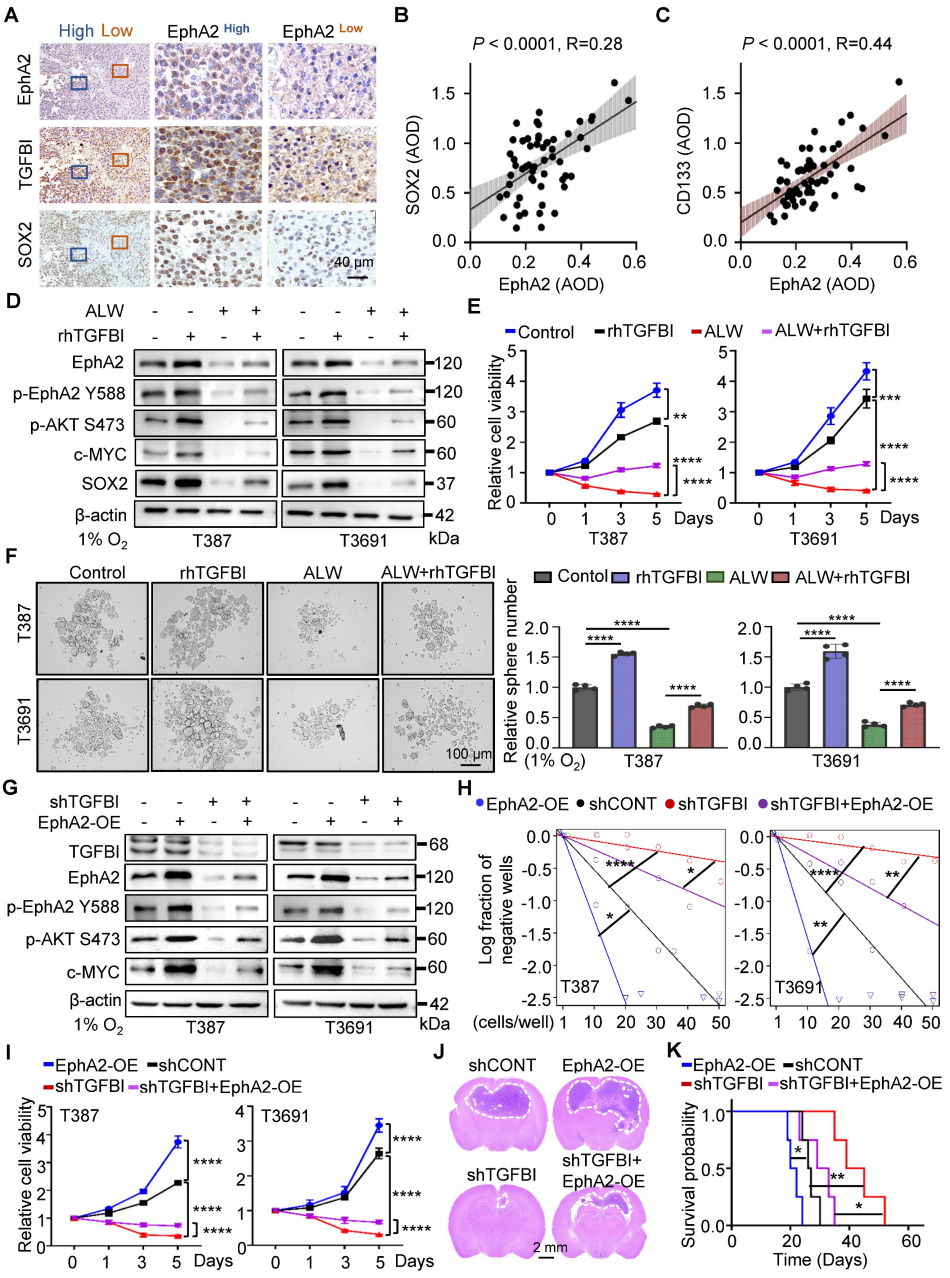
The binding of TGFBI to EphA2 promotes self-renewal and tumorigenesis of GSCs both *in vitro* and *in vivo*. (A) IHC staining showing TGFBI, EphA2, and SOX2 expression in the same human high- and low-expression GBM tissues. Scale bars: 40 μm. (B) IHC staining demonstrating the association between EphA2 and SOX2 and (C) CD133proteins in human gliomas. n = 58 (D) IB of AKT-c-MYC pathway and stem-associated proteins in the indicated GSCs. ALW, ALW-Ⅱ-41-27, an EphA2 inhibitor; rhTGFBI, recombinant human TGFBI protein. (E) Cell titer assay result of indicated GSCs. n = 6 (F) Bright-field microscopy (left) showing the tumorsphere formation of indicated GSCs under hypoxia. The quantification of relative tumorsphere number is shown on the right. n = 4; Scale bars: 100 μm. (G) IB of AKT-c-MYC pathway proteins in the indicated GSCs. OE, overexpression (H) Limiting dilution assays of the indicated GSCs. (I) Cell titer assay results of indicated GSC (n = 6). (J) H&E staining of brain sections from mouse xenograft. Scale bars: 2 mm (K) Kaplan-Meier survival curve of mice bearing indicated GSCs. n = 5; Data are presented as the mean ± SD. *P < 0.05; **P < 0.01; ****P < 0.0001.

Our study provides compelling evidence for the critical role of TGFBI in maintaining self-renewal and tumorigenesis of GSCs through stabilizing of EphA2. To evaluate this possibility, we initially examined the impact of EphA2 on GSCs. We observed a positive correlated between EphA2 and SOX2, except for TGFBI, in the same region of continuous human GBM tissue specimens (Figure 6A). Moreover, IHC staining of human glioma samples confirmed a positive correlation between EphA2 and both SOX2 and CD133 (Figure 6B-C). To further validate the mechanism, we introduced exogenous recombinant human TGFBI protein (rhTGFBI) to evaluate its impact on EphA2 and AKT-c-MYC signaling pathway in GSCs under hypoxic conditions. Besides, we integrated EphA2 inhibition, ALW-Ⅱ-41-27 (ALW), into our study. The results demonstrated that treatment with rhTGFBI significantly elevated EphA2 protein levels, activated the AKT-c-MYC signaling pathway, and enhanced the proliferation of GSCs under hypoxia. In contrast, treatment with ALW led to a decrease in stem cell characteristics. Moreover, rhTGFBI was observed to partially restore the signaling pathway and cellular phenotype impacted by EphA2 inhibition (Figure 6D-F).

Next, we investigated the capacity of EphA2 to restore the expression of downstream signaling molecules and the self-renewal ability of GSCs with TGFBI knockdown under hypoxia. As expected, the overexpression of EphA2 rescued the reduced expression of TGFBI downstream target genes and restored the sphere formation and cell viability in GSCs (Figure 6G-I). Moreover, *in vivo* xenografts demonstrated that EphA2 overexpression in shTGFBI mice (shTGFBI+EphA2-OE) accelerated tumor growth, as indicated by HE staining, and led to decreased mouse survival (Figure 6J-K). Collectively, these findings suggest that TGFBI can regulate the downstream AKT-c-MYC axis to maintain the self-renewal and tumorigenic capacity of GSCs under hypoxic conditions by interacting with EphA2.

## Discussion

TGFBI has been extensively studied in various malignancies, such as cholangiocarcinoma, renal cancer, ovarian cancer, colorectal cancer, and pancreatic cancer 8, 11, 20. Recognized primarily as an ECM protein pivotal in the invasive characteristics of highly malignant tumors, its role in supporting stem cell proliferation has recently gained considerable attention 21, 22. In our previous study, we demonstrated that the TAMs preferentially secrete TGFBI, which promotes GSC-driven tumor growth through integrin αvβ5-Src-Stat3 signaling 9.

In our current work, we have delved deeper into the role of TGFBI in GSCs. We observed minimal TGFBI expression in GSCs under normoxic conditions, with levels increasing in response to hypoxia and decreasing notably during GSC differentiation. This discovery helps to elucidate why M2-like TAMs secreted TGFBI exert a significant role in influencing GSCs in normoxic environments. The hypoxic microenvironment is widely acknowledged for enhancing the self-renewal and tumorigenic characteristics of GSCs, correlating with a poorer prognosis in patients 5, 14, 23. Consistently, we observed preferential expression of TGFBI in tumor cells of hypoxic regions in GBM specimen, suggesting hypoxia induced TGFBI expression. These observations suggest that TGFBI may employ novel mechanisms to regulate GSCs under hypoxic conditions.

Our mechanistic investigation unveiled that under hypoxic conditions, HIF1α binds to the specific binding motif in the TGFBI promoter within GSCs. The increased level of TGFBI subsequently bind to EphA2, triggering the downstream activation of the AKT-c-MYC signaling pathway. Meanwhile, TGFBI stabilizes EphA2 by inhibiting its ubiquitin-proteasome degradation pathway, which is consistent with findings from other studies 24-26. Knockdown of TGFBI resulted in a significant reduction in the self-renewal and tumorigenic capacity of GSCs via the TGFBI/EphA2 and AKT-c-MYC axis.

EphA2, a member of the tyrosine kinase family enriched in the PI3K/AKT pathway 27, has been shown to play a pivotal role in maintaining tumor cell stemness or promoting tumor growth through unconventional pathways 24-26, 28-31. Researches have indicated that the knockdown of EphA2 alone or in combination with EphA3 suppress the stem cell characteristics of GSCs via a kinase-independent mechanism 7, 32, which aligns with our findings.

While integrin has been reported as a binding receptor for TGFBI secreted by TAMs 9, 11, our study reveals that the upregulated expression of TGFBI predominantly binds to EphA2 in GSCs under hypoxia. Interestingly, Taniguchi H *et al.* have shown that bevacizumab can concurrently reduce EphA2 and TGFBI expression in colorectal cancer, suggesting a potential synergistic function of these molecules in malignant tumors 20. Following the identification of the binding between TGFBI and EphA2, the mechanism through which TGFBI regulates the degradation of EphA2 warrants consideration. As a transmembrane protein, EphA2 could be modified in cytosol. Chang Q 33 discovered that dasatinib inhibits the ligand-induced binding of EphA2 to the ubiquitin ligase Cbl, thus preventing the internalization and degradation of EphA2. Building upon these insights, we hypothesize that the interplay between TGFBI and EphA2 may alter the structure of EphA2, affecting its ubiquitin modification, internalization, and subsequent degradation.

Building upon our prior research on the impact of TGFBI secreted by TAMs on GSCs in normoxic conditions, our current investigations that integrate the critical tumor niche components of hypoxia and the immune microenvironment are essential. Meanwhile, TGFBI exerts its effects on GSCs through both autocrine and paracrine pathways, suggesting its dual influence elicited by microenvironmental cues via distinct predominant downstream pathways. These results emphasize the crucial role of TGFBI orchestrated within the intricate tumor microenvironment, potentially presenting a promising therapeutic target.

In conclusion, our results indicate that the upregulated TGFBI in GSCs plays a critical role in maintaining their stem cell characteristics, including self-renewal and tumorigenesis, within the hypoxic microenvironment. Therefore, interventions towards the TGFBI/EphA2 axis may offer a promising avenue for combatting GBM and improving clinical outcomes of patients.

## Supplementary Material

Supplementary figures and tables.

## Abbreviations

ALW: ALW-Ⅱ-41-27
AOD: average of density
CA9: carbonic anhydrase 9
CGGA: chinese glioma genome atlas
ChIP: chromatin immunoprecipitation
DGS: differentiated glioma stem cells
ECM: extracellular matrix
GBM: glioblastoma
GEO: gene expression omnibus
GSCs: glioma stem cells
HE: hematoxylin-eosin
IACUC: institutional animal care and use committee
IB: immunoblotting
IF: immunofluorescence
IHC: immunohistochemistry
RGD: arginine-glycine-aspartic
TAMs: tumor-associated macrophages
TCGA: the cancer genome atlas
TGFBI: transforming growth factor beta-induced protein
