# Supplementary material for: Hypoxia-induced TGFBI maintains glioma stem cells by stabilizing EphA2
Source: Theranostics. 2024 Sep 9;14(15):5778–92. doi: 10.7150/thno.95141 (PMC11426234; doi:10.7150/thno.95141)
Supplement: Supplementary file 1 — Supplementary figures and tables. [file thnov14p5778s1.pdf]

Supplemental Figures

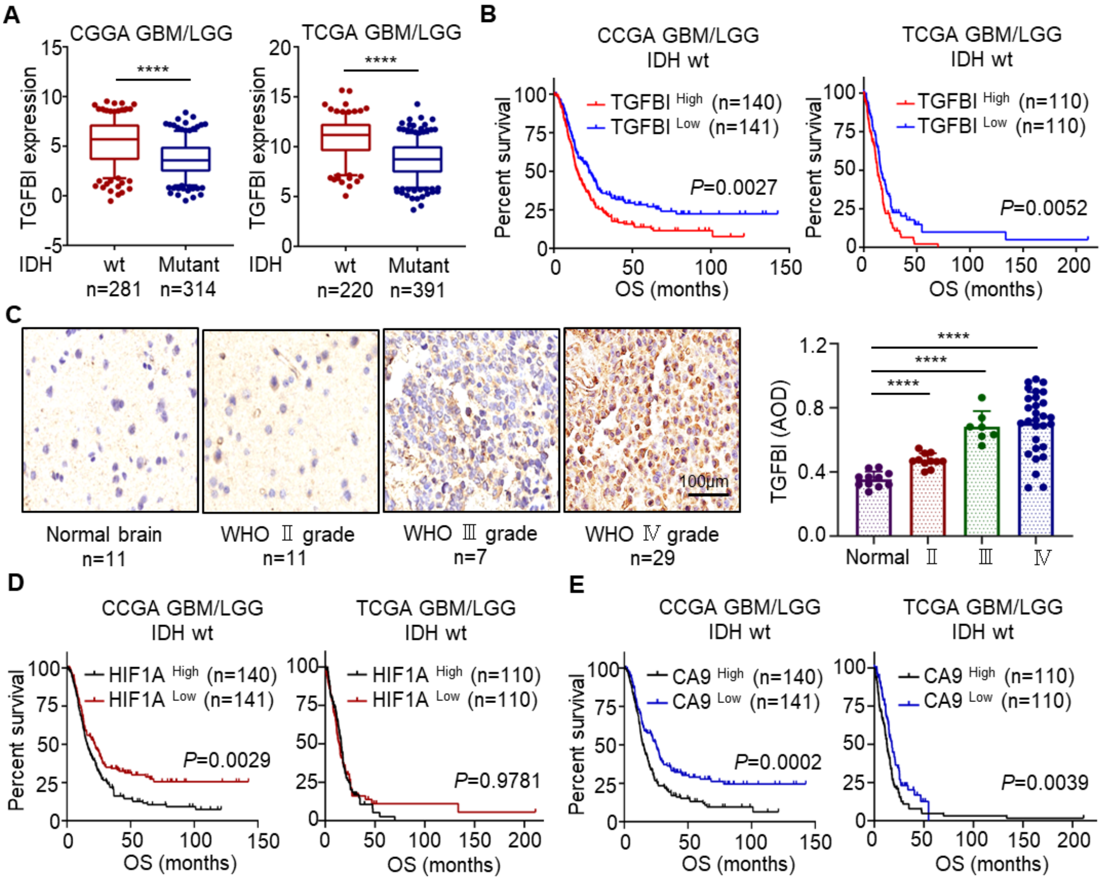

Figure S1. TGFBI expression correlates with glioma malignancy. (A) Box diagram reflecting TGFBI mRNA expression of the indicated cluster. Dataset: CGGA-GBM/LGG cohort, TCGA-GBM/LGG cohort (B) Kaplan–Meier survival curve of patients grouped by high- and low-TGFBI expression levels. (C) IHC staining of TGFBI in normal brain tissues and gliomas. Scale bars: 100 μm. Also shown is the quantification of the AOD of TGFBI. AOD, average density; ****P < 0.0001 (D) Kaplan–Meier survival curve of patients grouped by high- and low-HIF1α and (E) CA9 expression levels.

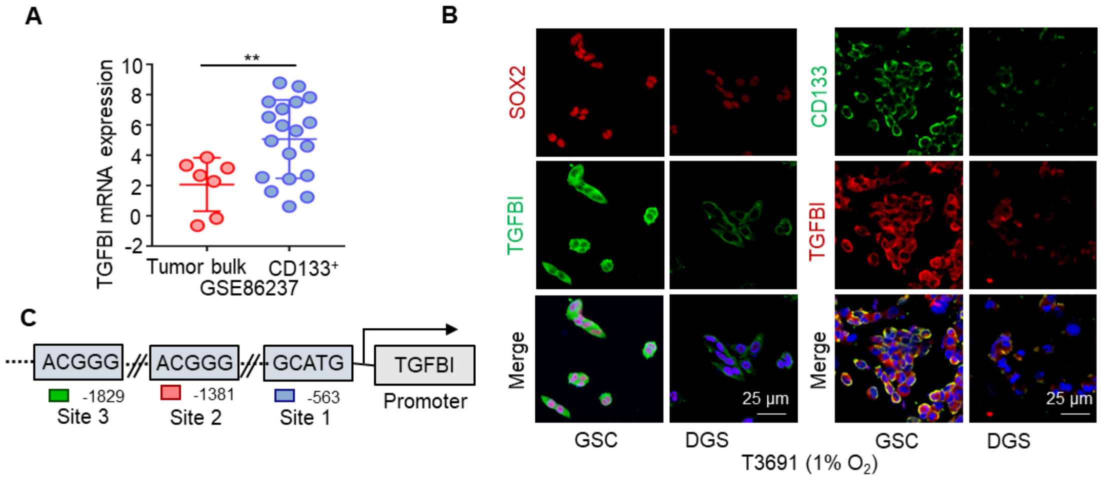

Figure S2. TGFBI is induced by HIF1α in GSCs under hypoxia. (A) Scatter plot showing the mRNA expression of TGFBI in tumor bulk and CD133$^+$ cells (GSE86237). (B) IF staining of TGFBI and two stem cell-associated markers (SOX2, CD133) in T3691 GSCs. (C) Schematic diagram showing three predicted HIF1α binding sites within the TGFBI promoter region. Data are presented as the mean ± SD. **P < 0.01.

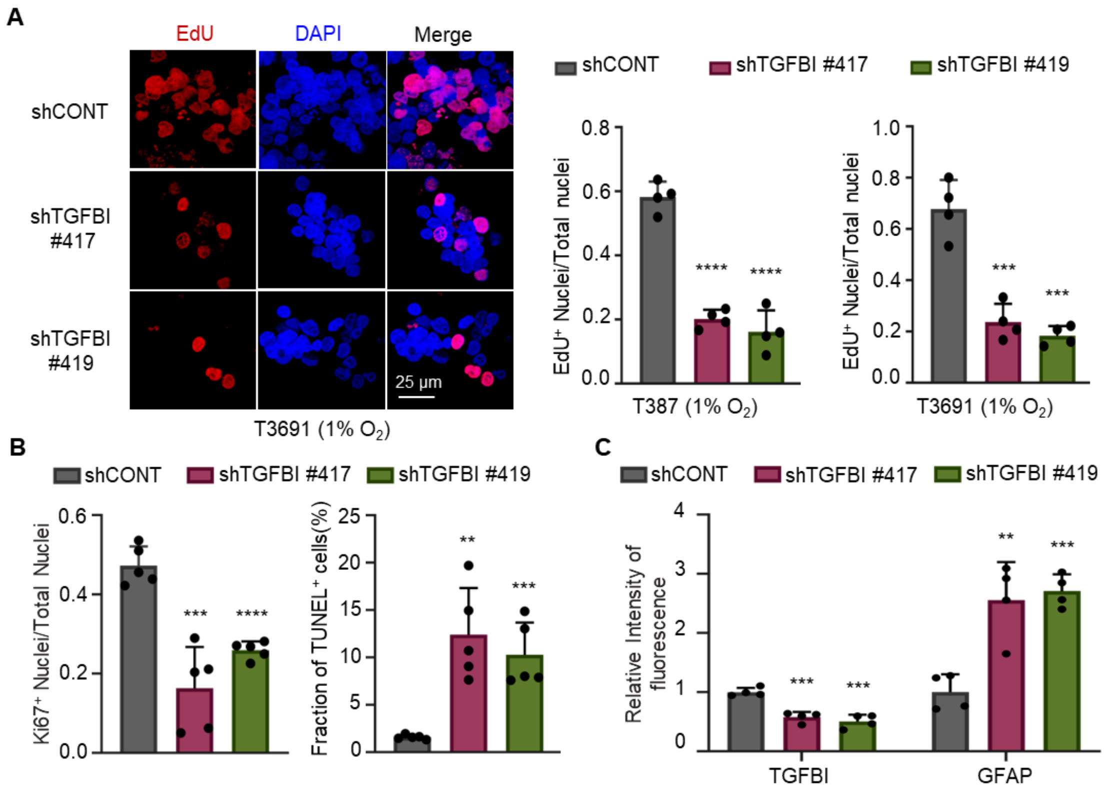

Figure S3. TGFBI play essential role in the self-renewal and tumorigenesis of GSCs. (A) IF image showing EdU incorporation in T3691 GSC tumorspheres. EdU is shown in red. Also shown is the quantification of the fraction of EdU^+ cells of indicated cells. n = 4; Scale bars: 25 μm. (B) The quantification of the fraction of $Ki67^+$ and $TUNEL^+$ cells in GBM xenografts. n = 5 (C) The quantification of the relative fluorescence intensity of TGFBI and GFAP in mouse xenografts. n = 4; Data are presented as the mean ± SD. **P < .01; ***P < .001; ****P < .0001.

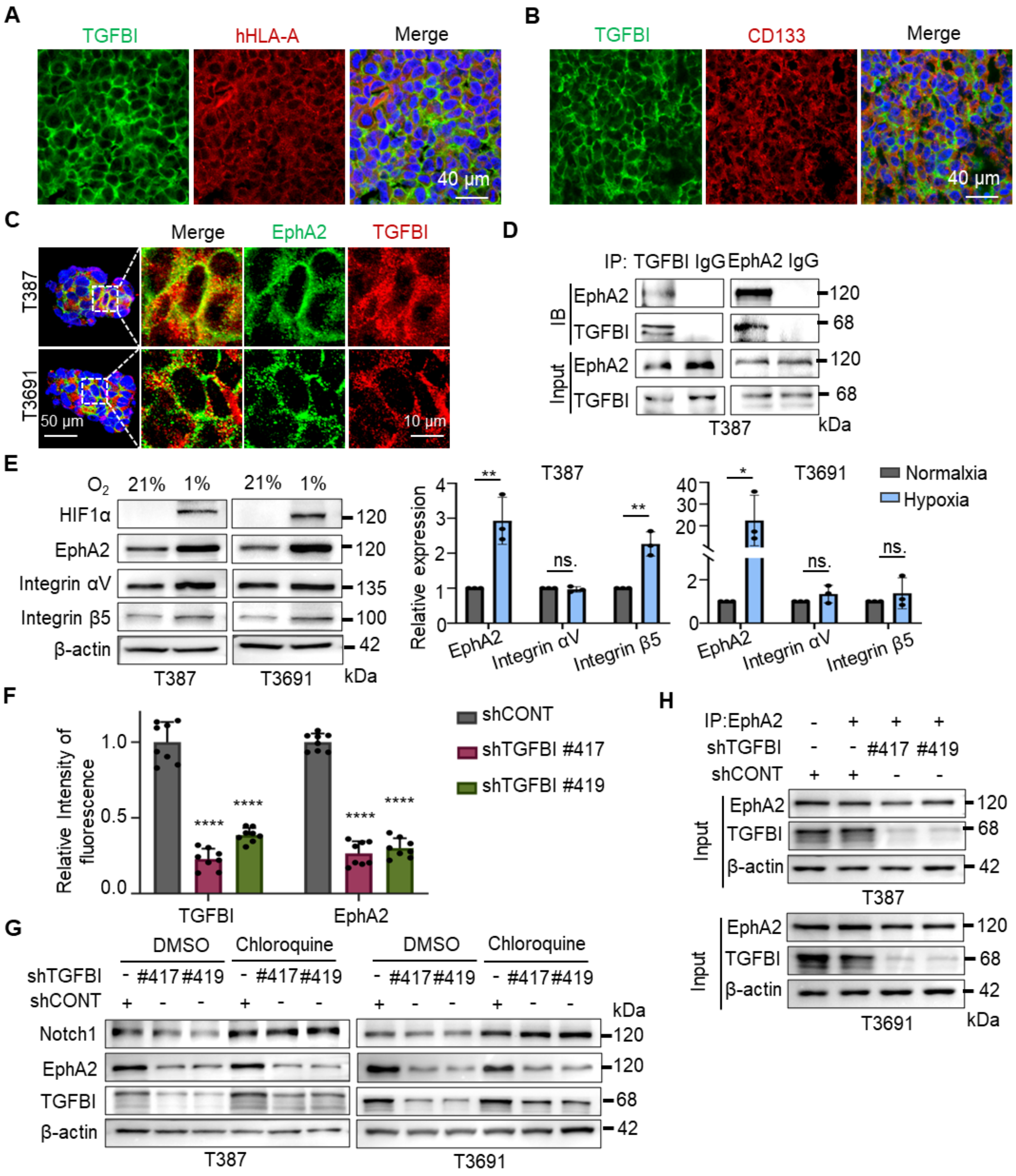

Figure S4. TGFBI binds to EphA2 and stabilizes it by inhibiting its proteasome degradation. (A) IF image of TGFBI and hHLA-A in mouse xenografts. hHLA-A, human HLA-A (B) IF image of TGFBI and CD133 in mouse xenografts. (C) IF image of TGFBI and EphA2 in GSC spheres. Scale bars: 50 μm, enlarged image: 10 μm (D) Co-IP of endogenous TGFBI and EphA2 in T387 GSCs under hypoxia. IgG served as a control. (E) IB of EphA2 and integrins in the indicated GSCs. Quantification of relative protein expression was provided on the right. n=3 (F) Quantification of the relative fluorescence intensity of TGFBI and EphA2 in the indicated mouse xenografts. n=8; Data are presented as the mean ± SD. ****P < 0.0001 (G) IB of EphA2 proteins in the indicated GSCs. Chloroquine, a lysosome inhibitor (H) Input for Co-IP of ubiquitin-EphA2 in the indicated GSCs.

Table S1. Detailed clinical information of glioma patients.

Patient No.	Gender	Age years	Grade	AOD					
				CD133	HIF1A	EPHA2	SOX2	C-MYC	TGFBI
1	male	63	N	0.209432	0.239455	0.120885	0.25261	0.142691	0.429167
2	male	47	4	0.608185	0.187662	0.167461	1.201126	0.286887	0.385811
3	male	33	N	0.411713	0.159646	0.1545	1.034036	0.246477	0.275275
4	male	44	N	0.263934	0.185342	0.158548	0.83617	0.315808	0.321759
5	male	55	4	0.628698	0.232074	0.295782	0.940246	0.415137	0.76621
6	male	63	4	0.488633	0.217969	0.189812	1.024079	0.527414	0.493867
7	male	58	4	1.212553	0.363702	0.364978	0.666862	0.604884	0.923638
8	female	47	N	0.380369	0.174527	0.267397	0.827736	0.715586	0.368772
9	female	44	2	0.471855	0.097551	0.339656	0.560454	0.153869	0.464834
10	male	65	N	0.345835	0.151298	0.260471	0.376234	0.264827	0.321397
11	male	68	4	0.724211	0.206485	0.227058	0.626464	0.775544	0.480371
12	male	35	4	0.948721	0.354474	0.222376	0.645788	1.155551	0.813952
13	female	42	4	0.990438	0.253543	0.354044	1.095725	0.5093	0.748459
14	male	30	4	0.81934	0.267449	0.146511	0.677616	0.425844	0.685725
15	female	35	4	1.07451	0.267917	0.520965	1.609054	0.591325	0.724777
16	female	53	4	0.766339	0.318907	0.241058	1.213581	0.765705	0.902628
17	male	48	2	0.807621	0.238126	0.25076	0.293985	0.466956	0.47562
18	male	47	2	0.768899	0.25754	0.262822	0.956526	0.377354	0.547108
19	female	44	3	0.621324	0.203353	0.305265	0.294096	0.646412	0.563745
20	male	46	N	0.270122	0.123311	0.107351	0.458731	0.302744	0.30778
21	female	43	2	0.618276	0.240555	0.141274	0.583753	0.665446	0.399336
22	male	60	N	0.524888	0.188396	0.131863	0.483766	0.44191	0.389532
23	male	65	4	0.824161	0.285199	0.24712	1.188283	0.678539	0.546518
24	female	56	4	0.71843	0.283632	0.207862	1.04413	0.844361	0.602433
25	female	50	4	1.615748	0.331947	0.571845	1.430964	0.908459	0.978135
26	female	50	4	1.149684	0.242746	0.44089	1.267347	0.837912	0.899654
27	male	50	3	0.542582	0.291594	0.437521	1.229996	0.520024	0.737178
28	female	42	4	0.77721	0.230384	0.344308	0.671831	0.421314	0.799399
29	male	45	4	0.602897	0.126204	0.278846	1.008153	1.076785	0.857327
30	male	44	N	0.374958	0.126496	0.168812	0.37706	0.312623	0.325148
31	male	46	4	0.406844	0.22803	0.225054	0.719324	0.526606	0.304432
32	female	51	2	0.547974	0.306768	0.246398	1.048459	0.804435	0.467654
33	male	65	4	1.116635	0.347715	0.266994	0.690812	0.658017	0.556706
34	male	62	4	1.279584	0.369306	0.396483	1.220092	1.130988	0.960289
35	female	36	4	0.516202	0.16159	0.178288	0.270128	0.368873	0.301024
36	female	35	4	0.944039	0.294767	0.33386	0.633343	0.790779	0.959058
37	male	30	3	0.77677	0.269081	0.300793	1.037392	0.590966	0.632953
38	female	16	2	0.464404	0.141045	0.170243	0.38311	0.372955	0.455142
39	female	26	4	0.560801	0.291889	0.422051	0.946074	0.787388	0.698466
40	male	43	2	0.530366	0.343245	0.201578	0.76369	0.723024	0.516504

41	male	46	4	0.731384	0.368417	0.217315	0.972388	0.838122	0.688519
42	female	38	4	0.949017	0.335157	0.249201	1.309399	0.760144	0.716353
43	female	38	4	0.939705	0.264221	0.297284	0.863892	0.698096	0.830233
44	female	54	3	0.597259	0.266161	0.231398	0.760884	0.590533	0.679172
45	male	46	3	0.614756	0.283909	0.255862	0.56533	0.59551	0.62243
46	female	48	2	0.77882	0.262482	0.166648	0.144858	0.371246	0.411685
47	male	31	3	0.741009	0.335312	0.247206	1.071817	0.42276	0.681156
48	male	55	4	0.628976	0.310384	0.144641	0.891773	0.530365	0.511519
49	female	37	N	0.424657	0.22667	0.18863	0.740819	0.294271	0.368756
50	male	53	N	0.36677	0.28207	0.196929	0.29521	0.351904	0.365904
51	male	47	4	0.686865	0.331295	0.323668	0.813878	0.640822	0.703143
52	male	31	4	0.745179	0.246093	0.398692	1.056336	0.4254	0.865275
53	female	9	2	0.503463	0.237632	0.177938	0.449761	0.488706	0.466424
54	female	50	N	0.502224	0.106963	0.256652	0.15135	0.439892	0.424025
55	male	2	3	0.594715	0.30818	0.275479	1.001746	0.741216	0.863787
56	male	34	4	0.490397	0.335516	0.211813	0.779768	0.614167	0.739501
57	male	17	2	0.372624	0.174471	0.163848	0.776065	0.276095	0.521496
58	male	45	2	0.50337	0.192029	0.241291	0.988321	0.438105	0.467338

AOD: Average of density= Cumulative optical density (IOD)/area; N, normal brain

Table S2. Sequence of qRT-PCR primers

Name		Primer sequence (5'-3')
β-actin	Forward	CATGTACGTTGCTATCCAGGC
	Reverse	CTCCTTAATGTCACGCACGAT
TGFBI	Forward	CTTCGCCCCTAGCAACGAG
	Reverse	TGAGGGTCATGCCGTGTTTC
EphA2	Forward	TGGCTCACACACCCGTATG
	Reverse	GTCGCCAGACATCACGTTG
HIF1A	Forward	AGTCTAGAGATGCAGCAAGATCTC
	Reverse	TTCCTCATGGTCACATGGATGAGT
ChIP primer, HIF1a	Forward	CCTCATGGGATGCTTCACCA
binding site 1	Reverse	GGAGTCTGTATACAGGGAGAAGG

ChIP primer, HIF1a	Forward	ATTAGTACCAAGGTGAGGACCC
binding site 2	Reverse	GTGATCCACAGAAACGCCAA
ChIP primer, HIF1a	Forward	GAAGACTGTGGCGAGGGAG
binding site 3	Reverse	AGGTTAAGTAAGCTGCGAGC
MYC	Forward	GTCAAGAGGCGAACACACAAC
	Reverse	TTGGACGGACAGGATGTATGC

Table S3. Mass Spectrometry Results of Potential TGFBI-Interacting Proteins.

Protein（IP only）	Abundances (Normalized)	DeltaM (ppm)
FLOT2	54998615.5	3.37
TNIK	22530246	2.66
GNB1	13182211	4.59
RAB10	3577150.75	1.46
EphA2	2367246	0.94
ANXA2	1510856.375	6.60
JUP	970029	0.73
FLG	102923.8125	8.19
